# Carbon and nitrogen isotopic variability in foxtail millet (*Setaria italica*) with watering regime

**DOI:** 10.1002/rcm.8615

**Published:** 2020-02-07

**Authors:** Emma Lightfoot, M. Cemre Ustunkaya, Natalia Przelomska, Tamsin C. O'Connell, Harriet V. Hunt, Martin K. Jones, Cameron A. Petrie

**Affiliations:** ^1^ McDonald Institute for Archaeological Research University of Cambridge Downing Street Cambridge CB2 3ER UK; ^2^ Department of Anthropology, National Museum of Natural History Smithsonian Institution Washington DC 20560 USA; ^3^ Center for Conservation Genomics Smithsonian Conservation Biology Institute National Zoo Washington DC 20008 USA; ^4^ Department of Archaeology University of Cambridge Downing Street Cambridge CB2 3DZ UK

## Abstract

**Rationale:**

Carbonised plant remains are analysed for reconstruction of past climates and agricultural regimes. Several recent studies have used C_4_ plants to address related questions, and correlations between modern C_4_ plant δ^13^C values and rainfall have been found. The millets were important food crops in prehistoric Eurasia, yet little is known about causes of isotopic variation within millet species. Previous research has shown there to be significant isotopic variation between millet accessions. Here we compare isotope ratios from plants grown under different watering regimes. This allows for a consideration of whether or not *Setaria italica* is a good proxy for environmental reconstruction.

**Methods:**

We compare stable isotope ratios of *Setaria italica* plants grown in a controlled environment chamber with different watering regimes. We compare the carbon isotope ratios of leaves and grains, and the nitrogen isotope ratios of grains, from 12 accessions of *Setaria italica*.

**Results:**

We find significant isotopic variability between watering regimes. Carbon isotope ratios are positively correlated with water availability, and on average vary by 1.9‰ and 1.7‰ for leaves and grains, respectively. Grain nitrogen isotope ratios also vary with watering regime; however, the highest isotope ratios are found with the 130‐mL watering regime.

**Conclusions:**

The carbon isotope ratios of *Setaria italica* are strongly correlated with water availability. However, the correlation is the opposite to that seen in studies of C_3_ plants. The difference in isotopic ratio due to watering regime is comparable with that seen between different accessions; thus distinguishing between changing varieties of *Setaria italica* and changing climate is problematic. In terms of grain nitrogen isotope ratios, the highest δ^15^N values were not associated with the lowest watering regime. Again, δ^15^N variation is comparable with that which would be expected from an aridity effect or a manuring effect, and thus distinguishing between these factors is probably problematic.

## 1 INTRODUCTION

Increasingly in recent years, stable isotope studies of charred plant remains have been used in archaeological research to answer questions about palaeoclimate and farming practices, as well as to improve our interpretations of human and animal isotope results.^1^, ^2^, ^3^, ^4^ Fundamental to this research is a sound understanding of the causes and magnitude of isotopic variation in plants. The causes of plant isotopic variation have been investigated using modern experimental studies, led by both plant scientists and archaeologists. For example, it has been shown that manuring can increase nitrogen isotope ratios by as much as 9‰ in cereals manured with cattle slurry.^5^ While most archaeological isotopic research on charred plant material has focused on C_3_ plants, most notably wheat and barley, increasing archaeological and isotopic research in China, Central Asia and Eastern Europe has highlighted the importance of millets (a generic term for all small‐grained cereals, which are typically found to be C_4_ plants) in the archaeological record.

Millets have various advantages over other major food crops in that they have a short growing season, relatively high nutritional value and high water use efficiency, and can grow on poor soil.^6^, ^7^, ^8^ Two species of millets are important for Eurasian prehistoric archaeology, foxtail and broomcorn millet (*Setaria italica* and *Panicum miliaceum*, respectively). While other C_4_ plants were probably available to prehistoric farmers, these species represent the only staple C_4_ crops distributed widely across Eurasia^9^ and, as such, are easily discernible in palaeodietary isotope studies of human and animal bone collagen. Both foxtail and broomcorn millet were domesticated in China before 5000 bc and spread across Eurasia to Europe by the middle Bronze Age (*c*. 1500 bc).^10^ Millet consumption has been shown both isotopically^11^ and archaeobotanically^12^ across prehistoric Eurasia. Carbonised millet grains therefore offer an opportunity to study palaeoclimate and farming practices in the past, as well as having the potential to provide baseline information for palaeodietary studies. Given the recent geographic expansion of isotopic archaeological applications, it is now timely to consider further the causes of isotopic variation in millet plants.

In a previous study,^13^ we reported on isotopic variation in different *Setaria italica* accessions grown in a controlled environment chamber. Our reasons for choosing *Setaria italica* included: its importance to archaeology; its high levels of intraspecific variability plus the recent sequencing of its genome (which facilitates analysis of the functional genetic variation underlying phenotypic variability)^14^, ^15^, ^16^; and its relatively short life cycle.^8^ That study showed significant isotopic variability within single leaves and panicles, and between leaves and panicles within the same plant. Carbon isotope ratios in leaves and grains varied by *c*. 2‰ between different accessions (a plant or grain sample, variety or population, collected from a particular area and kept in a gene bank for conservation, cultivation and research), while nitrogen isotope ratios in grains varied by *c*. 6‰. There was an average offset of 0.9‰ between leaf and grain carbon isotope ratios.

Here, we build on this previous research by characterizing carbon and nitrogen isotopic variation in *Setaria italica* plants subjected to different watering regimes. We grew four plants each of 12 *Setaria italica* accessions and subjected the plants to four different watering regimes (hereafter ‘experimental lines’). Control plants were also grown to characterise intra‐line variation due to environmental variation within the growth chamber and genetic variation within the line.

## 2 SCIENTIFIC BACKGROUND

### 2.1 C_4_ photosynthesis and isotope discrimination

There are two major photosynthetic pathways, C_3_ and C_4_, which use different methods of uptake of carbon dioxide from the atmosphere. C_4_ plants are more efficient in terms of water and nitrogen use than C_3_ plants, and have higher light use efficiencies above 25–30°C.^17^, ^18^ The majority of the world's plants use the C_3_ pathway, but several important crop plants are C_4_ including maize, sugar cane, sorghum and the millets. It is well established that multiple environmental and genetic factors affect the carbon isotope ratios of C_3_ plants.^19^, ^20^ These differences allow the use of carbon isotope ratios of charred plant remains to infer the environmental conditions under which they grew. C_4_ plants, however, are thought to be relatively insensitive to environmental factors and show less isotopic variability.^21^

Both photosynthetic pathways discriminate against ^13^C during the uptake of CO_2_, with C_4_ plants discriminating less than C_3_ plants. Isotopic discrimination in C_3_ plants is well understood and is largely controlled by the diffusion of CO_2_ through the stomata and the action of enzymes.^19^, ^22^ Isotopic discrimination in C_4_ plants is less well understood, but a theoretical basis has been presented.^22^, ^23^ The dissolution and hydration of CO_2_, and CO_2_ leakage from bundle sheath cells, as well as the stomatal and enzymatic components, are important. As primary fixation of CO_2_ occurs efficiently at lower concentrations than in C_3_ plants, C_4_ plants are less sensitive to the partial pressure of CO_2_ inside the leaf mesophyll and in the atmosphere. Discrimination should increase either through increases in the amount of CO_2_ that leaks out of the bundle sheath cell, or in the concentration of the enzyme phosphoenolpyruvate (PEP) carboxylase.^24^

There are three subtypes of C_4_ photosynthesis, relating to the different enzymes used to release CO_2_ in the bundle sheath cells. Although the reasons are not fully understood, these subtypes show small differences in δ^13^C values.^22^, ^25^, ^26^ *Setaria italica* uses the NADP‐ME (NADP‐malic‐enzyme) subtype, which has the highest δ^13^C values of the three subtypes when they are grown under controlled conditions.^25^, ^26^

Early compilations of plant carbon isotopic data showed that the range in C_3_ plants was larger than that of C_4_ plants,^27^ which could suggest that C_4_ plants are less affected by environmental parameters than C_3_ plants. However, there are isotopic differences across C_4_ plants based on, for example, bundle sheath anatomy.^26^, ^28^ Isotopic differences have also been shown between different varieties of maize (*Zea mays*; 2.2‰),^29^ sorghum (*Sorghum bicolor*),^30^ kleingrass (*Panicum coloratum*)*,* 31 and foxtail millet (*Setaria italica*).^13^, ^32^

Isotopic differences have been seen between photosynthetic and non‐photosynthetic tissue in C_4_ plants.^13^, ^32^, ^33^, ^34^ In terms of different chemical compounds, alkanes and lipids have been shown to have δ^13^C values that are 8–10‰ lower than those of bulk leaf matter in C_4_ species,^35^ and cellulose δ^13^C values tend to be higher than those of lignin).^36^, ^37^ Turning to environmental parameters, studies have shown relationships between C_4_ plant isotope ratios and light intensity,^38^, ^39^, ^40^ salinity,^41^ latitude,^32^ altitude^42^ and water availability,^38^, ^43^, ^44^ although the relationship in each instance is not always simple or linear.^32^, ^45^

In order to use C_4_ plants to reconstruct past environments and farming practices, we need to understand the isotopic variation within and between plants grown under the same conditions on an individual species level,^13^ and also characterise isotopic variation caused by multiple environmental parameters. This study adds to the limited body of literature available for *Setaria italica* by characterising the magnitude and strength of the relationship between water availability and plant δ^13^C values on an individual species level.

### 2.2 Nitrogen uptake and isotope discrimination

Nitrogen isotope ratios in plants are ultimately derived from the nitrogen taken up by the plant – atmospheric nitrogen (for nitrogen‐fixing plants) and other nitrogenous sources (NH_4_^+^ and NO_3_^−^). These sources have different nitrogen isotope ratios and the δ^15^N value of the plant depends upon the proportion of each of these components that is utilised, modified by the discrimination factors that occur for each.^46^, ^47^ The total soil δ^15^N values are controlled by: the composition of the soil^46^, ^47^; whether the soil is part of an open or closed system^48^, ^49^; the age, and therefore often depth, of the soil^50^, ^51^; climate, particularly rainfall^52^; salinity^53^; the amount and type of animal matter^54^, ^55^; and altitude.^56^ In general, soil δ^15^N values increase as ^15^N‐depleted mineral nitrogen compounds are lost due to nitrification, ammonia volatilisation and leaching.^57^

The nitrogen isotope ratios in plants are further modified from that of the source nitrogen by fractionation during nitrogen uptake, metabolism and distribution. This modification varies between species, depending on: the type of mycorrhiza^58^, ^59^; plant morphology and tissue type^60^; and root depth (due to variations in soil δ^15^N values with depth).^61^ Differences as large as 10‰ have been reported between co‐occurring species,^61^ and within‐species differences in nitrogen isotope ratios are seen with genotype in *Hordeum spontaneum*,^62^, ^63^ and *Setaria italica*.^13^ Differences in nitrogen isotope ratios also exist between different parts of the plant. Studies indicate that bread wheat (*Triticum aestivum*) grains have higher δ^15^N values than rachises^64^ and that plant leaves can have higher δ^15^N values than roots (tomato plant^65^ and komatsuna (Japanese spinach leaf, *Brassica campestris*),^66^ or vice versa (dwarfed mangroves).^67^

In terms of environmental parameters, plant δ^15^N values have been shown to vary with nutrient status and climate. When phosphorus is limiting and nitrogen is in excess, soil–plant fractionation is high; conversely, when phosphorus is in excess and nitrogen is limiting, soil–plant fractionation is low. However, these relationships are further complicated by mycorrhizal associations, foliar uptake of nitrogen, and so on.^67^, ^68^, ^69^ Studies have observed positive relationships between plant δ^15^N values and temperature, and negative relationships between plant δ^15^N values and annual precipitation or water availability on a community level^49^, ^52^, ^70^ (although studies on individual species often fail to find such relationships^5^, ^71^). These relationships are believed to relate to higher nitrogen loss in hot, arid environments than in colder, drier environments, which tend to conserve and recycle nitrogen.^49^ Nitrogen loss is associated with large fractionations, leaving the remaining soil nitrogen enriched in ^15^N and increasing δ^15^N values throughout the foodchain.^72^

In order to use δ^15^N values of plants to reconstruct past climates and farming practices, it is vital to understand the impact of water availability on the major crop species. Here we examine the effect of watering regime on the δ^15^N values of *Setaria italica* plants. It is particularly important to consider staple C_4_ plants in this manner, as in palaeodietary isotope studies of bone collagen, nitrogen isotope ratios are used to distinguish between C_4_ and marine foodchains. Where C_4_ plant nitrogen isotope ratios may be high due to aridity, distinguishing between C_4_ consumption and marine consumption may not be possible on the basis of bulk collagen isotope ratios alone. It is therefore important to understand the extent to which aridity can increase nitrogen isotope ratios in staple C_4_ plants.

## 3 MATERIALS AND METHODS

A total of 12 accessions of *Setaria italica* were analysed in this study, selected from a larger set of 360 accessions, for which grain was obtained from five germplasm banks: the National Institute of Agrobiological Sciences (NIAS, Tsukuba, Japan); the Leibniz‐Institut für Pflanzengenetik und Kulturpflanzenforschung (IPK Gatersleben, Germany); the N.I. Vavilov Institute of Plant Genetic Resources (VIR, St Petersburg, Russian Federation); the International Crops Research Institute for the Semi‐Arid Tropics (ICRISAT, Telangana, India); and the United States Department of Agriculture Agricultural Research Centre (USDA‐ARS, Washington DC, USA). Accessions are derived from samples of local varieties, originally collected from across Eurasia and parts of Africa and presumed to be adapted to the climatic conditions in the localities from which they were collected (details of the samples used in this study are given in the supporting information). Accessions are defined as such by the curating germplasm bank, and what constitutes a distinct accession will depend on the opinions of the original collector. The genetic diversity within accessions will further be shaped by the regeneration programme of the germplasm bank in which they are maintained, and thus will be variable between accessions.

In previous experimental work, randomly chosen grains from each of the 360 accessions were sown and plants grown to maturity, with panicles bagged to prevent cross‐pollination.^73^ The resulting S1 selfed grain (i.e. the progeny of a plant where the only pollen that could reach the stigma of the flowers was the pollen from the anthers of that same plant) was harvested, and these grains were used in the previous experiment and as part of the wider study of *Setaria italica* genetic diversity.^13^, ^73^ With the exception of one of the control accessions, the grains harvested as part of the initial study were used in the current experiment. The accessions grown here therefore represent seed derived from a second‐generation plant (S2 selfed grain), hereafter designated ‘lines’. Because *S. italica* is largely self‐pollinating, within‐plant heterozygosity is expected to be very low, and therefore the grain within a single line should be highly similar genetically. The 12 accessions chosen for this experiment were selected based on several pragmatic factors, that is ones that had a relatively short flowering time in the previous experiment and ones with good location information associated with them. Beyond these pragmatic factors, samples were chosen on the basis of their collection location, with our archaeological area of interest in mind (i.e. the Indus Civilisation). Grains for control line SIT0560 were taken from the sample originally sent by the germplasm bank in order to consider isotopic variation within the landrace.

The grains were sown in a Conviron controlled environment chamber (hereafter growth chamber) at the Sainsbury Laboratory, University of Cambridge (Cambridge, UK): 16 h of daylight, 350 μmol light level, 28°C day time temperature, 22°C night time temperature and 65% humidity. All plants were grown in a 1‐L pot in the same type of compost (40% peat, 40% soil, 20% grit with fertiliser, supplied by the Sainsbury Laboratory).

For each of the 12 experimental lines, four replicate plants were grown and subjected to a different watering regime, which commenced at germination. Each plant was watered with tap water every 2 days: 50 mL for replicate A; 100 mL for replicate B; 130 mL for replicate C; and 300 mL for replicate D. These watering regimes were calculated based upon minimum and maximum water requirements as indicated in the literature (i.e. minimum 300 mm, optimum 400–600 mm and maximum 4000 mm annual rainfall),^74^ based on a 13‐cm diameter pot size and a growing time of 120 days (which in reality proved to be an underestimate). Watering regime D was originally expected to be 500 mL every 2 days; however, this was reduced to 300 mL as that was the maximum amount that would reasonably fit into each plant pot. Nevertheless, the regime D plants had an excess of water, and it can be assumed that they were waterlogged at points during the experiment, although the redox potential of the soil was not quantified. The excess water remaining in the tray was discarded before each watering session. Two sets of control plants were also grown, with six replicates each, and watered using watering regime C. The control plants were chosen from the accessions used in the experimental treatments (i.e. they have the same accession codes, and are distinguished by the prefix ‘control’, below). The six SIT0555 control replicates were grown from S2 selfed grain, and reflect within‐line and within‐growth chamber variation (controlling for potential edge effects and so on). The six SIT0560 control replicates were grown from the seed originally sent from the germplasm and represent within‐accession and within‐growth chamber variation.

The plants were grown on five trays in one area of the growth chamber, with one tray per watering regime and one tray for the two sets of control plants. Each tray held 12 pots, hence 12 experimental lines and 6 plants for each of the control lines. The plants were rotated within their trays and the trays were also rotated when watered.

The plants were harvested when the plant dried out (despite continued watering), or after 6 months, whichever was earlier. At harvest, the plants were separated into panicles, stems and roots, and stored in 50‐mL transport tubes and zip lock bags. For the roots, as much (wet) soil was washed from the root ball as possible. All samples and sample types were dried in an oven (*c*. 40°C). As much (dry) soil was manually removed from the roots as possible before all sample types were weighed.

Following the protocol established in our previous experiment,^13^ leaves were chopped by hand and ground in a Retsch TissueLyser (Qiagen, Manchester, UK). For grains at least 30 grains were taken and ground together by hand for analysis. For carbon isotope analysis, the sample size analysed for leaf and grain was 0.8–1.0 mg. For nitrogen isotope analysis of grains, the sample size was calculated for each plant based on the percentage nitrogen values obtained in the carbon isotope analysis of the grain, typically 2–4 mg.

Samples were analysed at the Godwin Laboratory, University of Cambridge, using an elemental analyser (Costech, Valencia, CA, USA) coupled in continuous‐flow mode to a Delta V isotope ratio mass spectrometer (ThermoFinnigan, Bremen, Germany). Carbon and nitrogen stable isotope ratios are expressed as delta values (e.g. δ^13^C values) on the VPDB and AIR scales for carbon and nitrogen, respectively.^75^, ^76^, ^77^ Repeated measurements on international and in‐house standards (alanine: L‐alanine, Honeywell Fluka, Bucharest, Romania; caffeine: IAEA‐600, IAEA, Vienna, Austria; caffeine: Elemental Microanalysis, Okehampton UK; nylon: Nylon 6, Sigma‐Aldrich, Gillingham, UK; and protein 2: Protein standard OAS, Elemental Microanalysis) showed that the analytical error was less than <0.2‰ for carbon and <0.25‰ for nitrogen. Samples were run in triplicate. The reproducibility across the triplicate analyses (generally <0.2‰) indicates that the samples were homogenised well.

Statistical analyses were performed using Rstudio version 1.0.143.^78^ The code and data file are available in the supporting information. The data were tested for normality using histograms and Shapiro–Wilk tests, for equality of variance using Levene's tests, and sphericity using Mauchly tests, where appropriate. The statistical tests used were repeated measures analysis of variance (ANOVA) or Friedmans tests (with post‐hoc tests), Spearman's rho and an independent samples t test.

## 4 RESULTS

The full dataset is given in the supporting information.

### 4.1 Leaf carbon isotope variation

**Figure 1:**
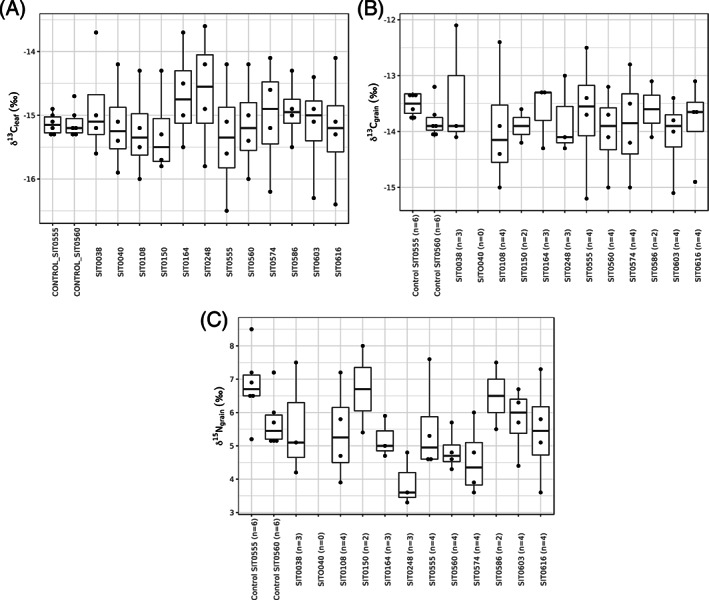
Boxplots showing A, δ^13^C_leaf_; B, δ^13^C_grain_; and C, δ^15^N_grain_ values, split by line

The δ^13^C_leaf_ results are summarised by line in Table 1 and shown in Figure 1A. The two control lines have a δ^13^C_leaf_ standard deviation of 0.2‰ each, a range of 0.4‰ and 0.6‰ (SIT0555 and SIT0560, respectively), and an interquartile range of 0.2‰ each. The experimental lines (*n* = 12, four watering regimes per line) have a mean δ^13^C_leaf_ range of 1.9‰ across the four regimes, with a mean standard deviation of 0.8‰ and a mean interquartile range of 0.7‰. The minimum within‐line δ^13^C_leaf_ range is 1.2‰ and the maximum is 2.3‰.

**Table 1:** Summary statistics of δ^13^C_leaf_ data, split by line

Line	**δ** ^**13**^ **C**_**leaf**_ **values**						
	n	Mean	Standard deviation	IQR	Minimum	Maximum	Range
CONTROL_SIT0555	6	−15.1	0.2	0.2	−15.3	−14.9	0.4
CONTROL_SIT0560	6	−15.1	0.2	0.2	−15.3	−14.7	0.6
SIT0038	4	−14.9	0.8	0.6	−15.6	−13.7	1.9
SIT0040	4	−15.2	0.7	0.7	−15.9	−14.2	1.7
SIT0108	4	−15.3	0.7	0.6	−16.0	−14.3	1.7
SIT0150	4	−15.3	0.7	0.6	−15.8	−14.3	1.5
SIT0164	4	−14.7	0.8	0.8	−15.5	−13.7	1.8
SIT0248	4	−14.6	1.0	1.1	−15.8	−13.6	2.3
SIT0555	4	−15.4	1.0	1.0	−16.5	−14.2	2.3
SIT0560	4	−15.2	0.8	0.7	−16.0	−14.2	1.9
SIT0574	4	−15.0	0.9	1.0	−16.2	−14.1	2.2
SIT0586	4	−14.9	0.5	0.4	−15.5	−14.3	1.2
SIT0603	4	−15.2	0.8	0.6	−16.3	−14.4	2.0
SIT0616	4	−15.2	0.9	0.7	−16.4	−14.1	2.3

**Figure 2:**
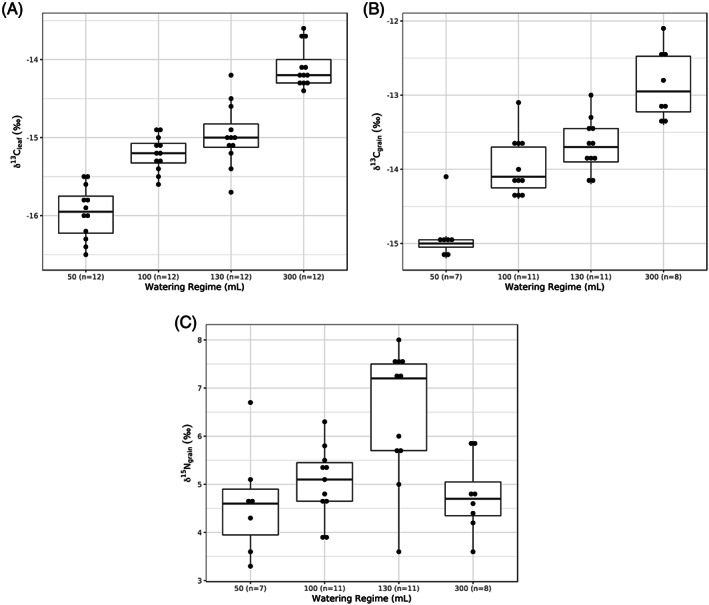
Boxplots showing A, δ^13^C_leaf_; B, δ^13^C_grain_; and C, δ^15^N_grain_ values, split by watering regime

**Figure 3:**
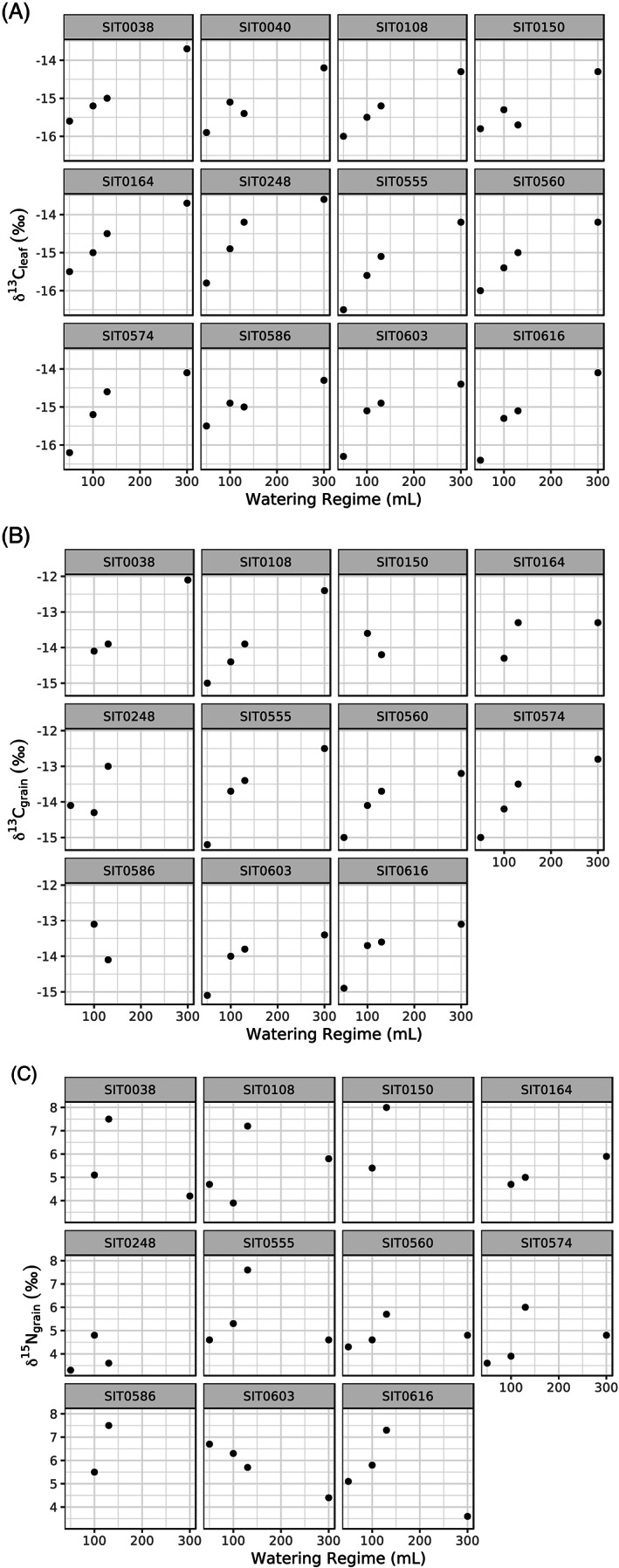
Scatter plots showing A, δ^13^C_leaf_; B, δ^13^C_grain_; and C, δ^15^N_grain_ values versus watering regime, split by line

The δ^13^C_leaf_ results are summarised by watering regime in Table 2 and shown in Figure 2A. The table indicates differences in δ^13^C_leaf_ values that are statistically significant (Friedman chi‐squared = 33.3, df = 3, *p* <0.001), with post‐hoc comparisons indicating that the δ^13^C_leaf_ values of the plants grown under watering regime A (50 mL) were significantly different from those from watering regimes C (150 mL) and D (300 mL). The results also show that the δ^13^C_leaf_ values of the plants grown under watering regime B (100 mL) were different from those from watering regime D (300 mL). The δ^13^C_leaf_ values are positively correlated with the watering regime (*r*_*s*_ = 0.88, S = 2102.6, *p* <0.001). When one considers each experimental line individually, most lines (9 of 12) follow this pattern of increasing δ^13^C_leaf_ values as the amount of water given increases (Figure 3A). There are three exceptions – SIT0040, SIT0150 and SIT0586.

**Table 2:** Summary statistics of δ^13^C_leaf_ data, split by watering regime

Watering regime	δ^**13**^ **C**_**leaf**_ **values**						
	n	Mean	Standard deviation	IQR	Minimum	Maximum	Range
A	12	−16.0	0.3	0.5	−16.5	−15.5	0.9
B	12	−15.2	0.2	0.3	−15.6	−14.9	0.7
C	12	−15.0	0.4	0.3	−15.7	−14.2	1.5
D	12	−14.1	0.3	0.3	−14.4	−13.6	0.8

### 4.2 Grain carbon isotope variation

The δ^13^C_grain_ results are summarised by line in Table 3 and shown in Figure 1B. Control line SIT0555 (*n* = 6) has a δ^13^C_grain_ range of 0.5‰, a standard deviation of 0.2‰, and an inter‐quartile range of 0.4‰. Control line SIT0560 (n = 6) has a δ^13^C_grain_ range of 0.9‰, a standard deviation of 0.3‰ (although one plant has an outlying δ^13^C_grain_ value of 13.2‰, with this sample removed the range is 0.4‰), and an interquartile range of 0.2‰. The experimental lines (*n* = 11, four regimes per line, although not all plants produced grain) have a mean δ^13^C_grain_ range of 1.7‰ across the watering regimes, with a mean standard deviation of 0.8‰ and a mean interquartile range of 0.7‰. If only the experimental lines which produced panicles under all four watering regimes are included (*n* = 6), the mean δ^13^C_grain_ range is 2.0‰ across the four regimes, the mean standard deviation is 0.9‰, and the mean interquartile range is 0.8‰. The minimum within‐line δ^13^C_grain_ range is 0.6‰ (SIT0150, only two plants produced panicles) and the maximum is 2.6‰ (SIT0555, all four plants produced panicles).

**Table 3:** Summary statistics of δ^13^C_grain_ data, split by line

Line	δ^**13**^ **C**_**grain**_ **values**						
	n	Mean	Standard deviation	IQR	Minimum	Maximum	Range
CONTROL_SIT0555	6	−13.5	0.2	0.4	−13.8	−13.3	0.5
CONTROL_SIT0560	6	−13.8	0.3	0.2	−14.1	−13.2	0.9
SIT0038	3	−13.4	1.1	1.0	−14.1	−12.1	2.0
SIT0108	4	−13.9	1.1	1.0	−15.0	−12.4	2.6
SIT0150	2	−13.9	0.4	0.3	−14.2	−13.6	0.6
SIT0164	3	−13.6	0.5	0.5	−14.3	−13.3	0.9
SIT0248	3	−13.8	0.7	0.7	−14.3	−13.0	1.4
SIT0555	4	−13.7	1.1	0.9	−15.2	−12.5	2.6
SIT0560	4	−14.0	0.8	0.8	−15.0	−13.2	1.8
SIT0574	4	−13.9	1.0	1.1	−15.0	−12.8	2.3
SIT0586	2	−13.6	0.7	0.5	−14.1	−13.1	1.0
SIT0603	4	−14.1	0.7	0.6	−15.1	−13.4	1.7
SIT0616	4	−13.8	0.7	0.5	−14.9	−13.1	1.8

The δ^13^C_grain_ results are summarised by watering regime in Table 4 and shown in Figure 2B. The table indicates differences in δ^13^C_grain_ values across the four watering regimes that are statistically significant (F = 68.43, df = 3, *p* <0.001), with post‐hoc comparisons indicating that the δ^13^C_grain_ values of the plants grown under all the watering regimes were different, with the exception that plants under watering regimes B (100 mL) and C (130 mL) were not statistically different. The δ^13^C_grain_ values are positively correlated with the watering regime (*r*_*s*_ = 0.83, S = 1460.1, *p* <0.001). Considering each line individually, most lines (8 of 11) follow a pattern of increasing δ^13^C_grain_ values with greater amount of water given (Figure 3B). There are three exceptions – SIT0150, SIT0248 and SIT0586.

**Table 4:** Summary statistics of δ^13^C_grain_ data, split by watering regime

Watering regime	δ^**13**^ **C**_**grain**_ **values**						
	n	Mean	Standard deviation	IQR	Minimum	Maximum	Range
A	7	−14.9	0.4	0.1	−15.2	−14.1	1.0
B	11	−14.0	0.4	0.5	−14.4	−13.1	1.3
C	11	−13.7	0.4	0.5	−14.2	−13.0	1.2
D	8	−12.9	0.5	0.7	−13.4	−12.1	1.3

Comparing δ^13^C_leaf_ and δ^13^C_grain_ values shows that all the grains have higher δ^13^C values than the leaves from the same plant, with a mean difference of 1.3‰ (range from 0.4 to 2.0‰; t (83)= −7.58, *p* <0.001).

### 4.3 Grain nitrogen isotope variation

The δ^15^N_grain_ results are summarised by line in Table 5 and shown in Figure 1C. Control line SIT0555 (*n* = 6) has a δ^15^N_grain_ range of 3.3‰, a standard deviation of 1.1‰ (although two plants have outlying δ^15^N_grain_ values, with these samples removed the range is 0.7‰), and an interquartile range of 0.6‰. Control line SIT0560 (*n* = 6) has a δ^15^N_grain_ range of 2.2‰, a standard deviation of 0.8‰ (although one plant has an outlying δ^15^N_grain_ value of 7.2‰, with this sample removed the range is 0.9‰), and an interquartile range of 0.7‰. The experimental lines (*n* = 11, four regimes per line, although not all plants produced grain) have a mean δ^15^N_grain_ range of 2.4‰ across the regimes, with a mean standard deviation of 1.2‰ and a mean interquartile range of 1.1‰. If only the experimental lines which produced panicles under all four watering regimes are included (*n* = 6), the mean δ^15^N_grain_ range is 2.7‰ across the four regimes, with a mean standard deviation of 1.2‰ and a mean interquartile range of 1.1‰. The minimum within‐line δ^15^N_grain_ range is 1.2‰ (SIT0164, three plants with panicles) and the maximum is 3.7‰ (SIT0616, all four plants produced panicles).

**Table 5:** Summary statistics of δ^15^N_grain_ data, split by line

Line	δ^**15**^ **N**_**grain**_ **values**						
	n	Mean	Standard deviation	IQR	Minimum	Maximum	Range
CONTROL_SIT0555	6	6.8	1.1	0.6	5.2	8.5	3.3
CONTROL_SIT0560	6	5.7	0.8	0.7	5.1	7.2	2.2
SIT0038	3	5.6	1.7	1.6	4.2	7.5	3.3
SIT0108	4	5.4	1.5	1.7	3.9	7.2	3.4
SIT0150	2	6.7	1.8	1.3	5.4	8.0	2.6
SIT0164	3	5.2	0.6	0.6	4.7	5.9	1.2
SIT0248	3	3.9	0.8	0.7	3.3	4.8	1.4
SIT0555	4	5.5	1.4	1.2	4.6	7.6	3.0
SIT0560	4	4.8	0.6	0.5	4.3	5.7	1.4
SIT0574	4	4.6	1.1	1.3	3.6	6.0	2.4
SIT0586	2	6.5	1.4	1.0	5.5	7.5	2.0
SIT0603	4	5.8	1.0	1.1	4.4	6.7	2.4
SIT0616	4	5.4	1.5	1.4	3.6	7.3	3.7

The δ^15^N_grain_ results are summarised by watering regime in Table 6 and shown in Figure 2C. There are statistical differences in the δ^15^N_grain_ values between the four watering regimes (F = 5.557, df = 3, *p* = 0.009), with post‐hoc tests showing that plants under watering regime C (130 mL) are statistically different from those under watering regimes A (50 mL) and D (300 mL) and marginally different from those under watering regime B (100 mL). There is no correlation between the δ^15^N_grain_ values and the amount of water given (*r*_*s*_ = 0.19, S = 6852.8, *p* = 0.266). Considering each line individually, most lines (8 of 11) follow the pattern of having a high δ^15^N_grain_ value for watering regime C (130 mL) and relatively similar δ^15^N_grain_ values for watering regimes A (50 mL), B (100 mL), and D (300 mL) (Figure 3C). There are three exceptions: SIT0164 (plants under watering regime D have the highest δ^15^N_grain_ values); SIT0248 (plants under watering regime B have the highest δ^15^N_grain_ values); and SIT0603 (plants under watering regime A have the highest δ^15^N_grain_ values).

**Table 6:** Summary statistics of δ^15^N_grain_ data, split by watering regime

Watering regime	δ^**15**^ **N**_**grain**_ **values**						
	n	Mean	Standard deviation	IQR	Minimum	Maximum	Range
A	7	4.6	1.1	0.9	3.3	6.7	3.4
B	11	5.0	0.7	0.8	3.9	6.4	2.5
C	11	6.5	1.4	1.8	3.6	8.0	4.4
D	8	4.8	0.8	0.7	3.6	5.9	2.3

## 5 DISCUSSION

### 5.1 Carbon isotopic variation

The isotopic patterning in leaf and grain is similar across the different watering regimes. Both the control lines have a smaller interquartile range of δ^13^C_leaf_ values than any of the experimental lines and a smaller interquartile range of δ^13^C_grain_ values than most of the experimental lines. Only experimental line SIT0150 has a smaller IQR than control line SIT0555. However, SIT0150 only produced two panicles and clearly failed to thrive under the conditions in the growth chamber, probably due to some or all of the relatively long day length, the temperature and the humidity as well as the water availability. We therefore conclude that within‐line isotopic variation and any variation caused by position in the growth chamber is less than the carbon isotopic variation caused by the watering regime.

The interquartile range of the two control lines is similar for both δ^13^C_leaf_ and δ^13^C_grain_ values. Given that control line SIT0555 was grown from S2 selfed seed while control line SIT0560 represents grain from the original accession (i.e. grain derived directly from the germplasm bank) this is surprising. It is currently unclear if this similarity simply reflects a sample size effect, and the outlying plant (SIT0560‐1) reflects diversity pertaining to the original (i.e. field‐collected) landrace, or if the true variability within these two control lines is indeed similar. In this latter scenario, it further remains unclear whether or not the replication, sampling for export and so on by the germplasm bank have led to homogenisation of this landrace or if the assumption that landraces will show relatively high genetic, phenotypic and isotopic variation is, in this case at least, untrue.

Comparing the δ^13^C_leaf_ and δ^13^C_grain_ results by watering regime clearly shows that the amount of water given to the plants had a strong effect on the carbon isotope ratios for both leaves and grains. In fact, the watering regime accounts for over 80% of the variation in δ^13^C values (*r*_*s*_ = 0.88 and 0.83 for leaf and grain, respectively). In theory, therefore, *Setaria italica* carbon isotope ratios can be used for the reconstruction of water availability in the present and also the past (provided, of course, that the other potential problems are resolved, such as preservation of the primary isotope signal, removal of contamination, and so on). There are, however, two problems which are likely to make this difficult in practice.

First, the mean difference in carbon isotope ratios between watering regime A (50 mL) and watering regime D (300 mL) is only 1.9‰ for leaves and 1.7‰ or 2.0‰ for grains (all experimental lines or those experimental lines which produced panicles under all watering regimes, respectively). This is similar variation to that seen between 29 different lines grown under uniform conditions in our previous experiment (*c*. 2‰),^13^ which indicates that it is not possible to distinguish between a genetic change and variation in water availability on the basis of carbon isotope analysis alone. If one found a difference of up to 2‰ between two groups of charred *Setaria italica* seeds, it would not be possible to distinguish between differences being caused by genetic variation (whether through drift or the planting of a new variety of *Setaria italica*) on the one hand, and changing water availability (and therefore climate or irrigation) on the other. While it may be possible to induce higher variability by using different watering regimes, we would argue that this would be difficult. Watering regime D (300 mL) resulted in the plants growing in saturated soil, with standing water in the trays; these plants were grown in an excess of water and increasing the amount of water given even further should not have any additional effect. At the parched end of the spectrum, watering regime A (50 mL) had the lowest successful production of grain and, while reducing the water given may increase isotopic variability, it would also probably reduce the number of plants that produced grain for analysis.

A second problem is the nature of the correlation between water availability and carbon isotopic ratios in millet. In most C_3_ plants, there is a negative relationship between water availability and carbon isotopic ratios, related to water use efficiency (WUE).^22^ In this study we found a positive correlation between the carbon isotope ratio and the amount of water given, as was also found by An and colleagues^32^ although only for plants grown in areas with less than 450 mm of rainfall a year. It has been known in the plant science community for some time that the δ^13^C value of a C_4_ plant can increase or decrease in response to drought,^22^, ^79^ depending upon the amount of CO_2_ that leaks out of the bundle‐sheath cells (leakiness, ɸ). Leakiness is determined by the bundle sheath's conductance to CO_2_ and the CO_2_ gradient between the bundle‐sheath and mesophyll cells, which is itself determined by the activities of PEP carboxylase and Rubisco.^79^ Although the underlying mechanisms that alter leakiness are not well understood,^80^ under most environmental conditions, leakiness is relatively low (<0.37) and the δ^13^C value will decrease with increasing water availability. However, this pattern is not the case for our samples. The δ^13^C value of a C_4_ plant may therefore either increase or decrease with increasing water availability. While there may be scenarios where determining *change* is the primary aim, in most scenarios the *direction* of said change towards higher or lower water availability is probably the purpose of the study. The use of C_4_ plants to study water availability in the past and present, therefore, seems to be of limited potential.

While not the aim of this study, we note that the mean difference in δ^13^C values between grains and leaves (1.3‰) is slightly higher than that seen in other studies.^13^, ^32^ As noted elsewhere, this pattern has implications for the interpretation of animal and human bone collagen isotope results, particularly where humans and animals eat different parts of the same plant.^13^

### 5.2 Nitrogen isotopic variation

The control lines have δ^15^N_grain_ interquartile ranges that are generally smaller than those of the experimental lines but for three experimental lines (SIT0164, SIT0248 and SIT0560) this is not the case. This pattern indicates that the variation caused by intra‐line differences and any variation caused by position in the growth chamber are, in some cases, as big as that caused by the watering regime.

The δ^15^N_grain_ variation within the two control lines is similar when the outliers are excluded and is more substantial in control line SIT0555 when the outliers are included. This pattern is the opposite to would be expected given that control line SIT0555 was grown from S2 selfed seed while control line SIT0560 represents grain from the original accession (i.e. grain derived directly from the germplasm bank). Nevertheless, this pattern indicates that the analysed landrace is not more diverse isotopically than the selfed lines.

Comparing the δ^15^N_grain_ results by watering regime indicates that while the watering regime does have an effect on plant nitrogen isotope ratios, this effect is not as expected. There is not a simple relationship between the nitrogen isotope ratio and the amount of water given, nor do the plants given the lowest amount of water have the highest nitrogen isotope ratios, as would be expected with an aridity effect.^81^, ^82^, ^83^ This finding indicates that *Setaria italica* grain δ^15^N values are not negatively correlated with water availability and, as such, they cannot be used as a palaeoclimate proxy in this way. It follows from this that aridity cannot simply be used to explain high human bone collagen δ^15^N values in populations consuming millet, as while aridity does affect plant δ^15^N values this is not necessarily in a predictable way (e.g. 84, 85). Rather, the data presented here suggests that, in relation to *Setaria italica* at least, high δ^15^N values are associated with well‐watered (but not over‐watered) plants. High nitrogen isotope ratios in both *Setaria italica* grains and human bone collagen from millet‐eating populations may therefore be indicative of optimal water availability rather than aridity.

The within‐line δ^15^N variation with watering regime reported here (mean = 2.7‰) is less than the variation seen between 29 different lines in our previous experiment (6‰).^13^ This is clearly problematic as, in the case of *Setaria italica* at least, increases in nitrogen isotope ratios could be related to genetic variation, aridity or manuring, amongst other factors. We would therefore recommend that plant isotope analysis is conducted in conjunction with other studies (such as grain morphometrics, weed seed analysis and other climate proxies) in order to provide a robust understanding of the past.

## 6 CONCLUSIONS

This study has shown that the carbon isotope ratios of *Setaria italica* are strongly correlated with water availability, but the correlation is the opposite to that seen in studies of C_3_ plants. The change in isotopic ratio due to watering regime is comparable with that seen due to change in accession. Thus, distinguishing between changing varieties of *Setaria italica* and changing climate is problematic. In terms of grain nitrogen isotope ratios, the highest δ^15^N values were not associated with the lowest watering regime, as would be expected if aridity were the cause of these high δ^15^N values. Again, the variation in δ^15^N values is comparable with that expected from an aridity effect or a manuring effect, and thus distinguishing between these factors is likely to be problematic. We suggest that in order to use the stable isotope ratios of archaeological *Setaria italica* grains to investigate past cultivation practices, these data are best used in conjunction with other lines of evidence.

## Supporting information

Data S1: Sample details

Data S2: Full dataset of *Setaria italica* watering experiment

Data S3: R code

Data S4: Supporting information
