# Supplementary material for: Carbon and nitrogen isotopic variability in foxtail millet (Setaria italica) with watering regime
Source: Rapid Commun Mass Spectrom. 2020 Feb 7;34(6):e8615. doi: 10.1002/rcm.8615 (PMC7050514; doi:10.1002/rcm.8615)
Supplement: Supplementary file 4 — Data S4: Supporting information [file RCM-34-e8615-s004.docx]

Supplementary Information 1: Sample details

Supplementary Information 2: Full dataset of *Setaria italica* watering experiment

Supplementary Information 3: R code
